# PDGFRA gene, maternal binge drinking and obstructive heart defects

**DOI:** 10.1038/s41598-018-29160-9

**Published:** 2018-07-23

**Authors:** Xinyu Tang, Johann K. Eberhart, Mario A. Cleves, Jingyun Li, Ming Li, Stewart MacLeod, Wendy N. Nembhard, Charlotte A. Hobbs

**Affiliations:** 1 Biostatistics Program, University of Arkansas for Medical Sciences, Arkansas Children’s Research Institute, Little Rock, 72202 USA; 2 Division of Birth Defects Research, Department of Pediatrics, College of Medicine, University of Arkansas for Medical Sciences, Arkansas Children’s Research Institute, Little Rock, 72202 USA; 3 Department of Molecular and Cell and Developmental Biology, Institute for Cellular and Molecular Biology and Institute for Neuroscience, University of Texas, Austin, 78712 USA; 4 Department of Epidemiology and Biostatistics, School of Public Health, Indiana University at Bloomington, Bloomington, 47405 USA

## Abstract

Obstructive heart defects (OHDs) are a major health concern worldwide. The platelet-derived growth factor (*PDGF*) genes are known to have regulatory functions that are essential for proper heart development. In a zebrafish model, *Pdgfra* was further demonstrated to interact with ethanol during craniofacial development. In this article, we investigated interactions between variants in *PDGF* genes and periconceptional alcohol exposure on the risk of OHDs by applying log-linear models to 806 OHD case and 995 control families enrolled in the National Birth Defects Prevention Study. The interactions between four variants in *PDGFA* and maternal binge drinking reached a nominal significance level. The maternal *T* allele of rs869978 was estimated to increase OHD risk among women who binge drink, while infant genotypes of rs2291591, rs2228230, rs1547904, and rs869978 may reduce the risk. Although none of these associations remain statistically significant after multiple testing adjustment and the estimated maternal effect may be influenced by unknown confounding factors, such as maternal smoking, these findings are consistent with previous animal studies supporting potential interactions between the *PDGFRA* gene and maternal alcohol exposure. Replication studies with larger sample sizes are needed to further elucidate this potential interplay and its influence on OHD risks.

## Introduction

More than 85% of congenital heart defects are thought to result from a complex interplay between maternal exposures, genetic susceptibilities, and epigenetic phenomena^1^. Among all congenital heart defects, right- and left-sided obstructive heart defects (OHDs) are a major health concern since they are the most lethal subtypes of congenital heart defects in infancy^2^. Although studies have investigated the interaction between maternal exposures and genetic variants on risk of congenital heart defects^3–8^, the impact of various gene-environment interactions during cardiogenesis remains largely unknown.

Alcohol exposure has been linked to the risk of congenital heart defects in both mice and human studies^9,10^. Webster *et al*. 1984 demonstrated that a relative short exposure to high doses of alcohol during pregnancy in mice can cause congenital heart defects^9^. Carmichael *et al*.^10^ reported that the risks of conotruncal heart defects associated with consuming five or more drinks per drinking occasion were 1.6 for less than once a week, and 2.4 for once a week or more^10^. However, recent lines of research suggested no association between alcohol exposure and congenital heart defects^11–14^. Analysis of the data from 7,076 mothers whose children were affected by congenital heart defects and 7,972 control mothers enrolled in the National Birth Defects Prevention Study (NBDPS) did not show statistically significant increased risks between maternal alcohol consumption and congenital heart defects^12^. Three recent meta-analyses also suggested that periconceptional alcohol exposure was not associated with congenital heart defects^11,13,14^. From these mixed findings, it is suspected that periconceptional alcohol exposure may indirectly impact the occurrence of congenital heart defects via gene-ethanol interactions.

Studies in animal models support the involvement of ethanol exposure in the genesis of congenital heart defects and demonstrate that this genesis is likely to be complex. In zebrafish, ethanol exposure at different stages results in differing heart defects^15,16^. Similarly, ethanol exposure during gastrulation results in cardiac defects in mouse^17^. In both of these models, the cardiac defects were rescued by folate administration, suggesting that the genesis of congenital heart defects following ethanol exposure in humans is a complex and multifactorial event. There is growing evidence that gene-ethanol interactions modulate the teratogenic effects of ethanol^18^, further adding complexity to ethanol-induced congenital heart defects. Animal models have given insight into some of these gene-ethanol interactions, often these ethanol-sensitive loci are genes governing the development of the organ system^18^.

Proper development of both the face and the heart requires the function of the receptor tyrosine kinase in platelet-derived growth factor receptor alpha (*PDGFRA*). Both mouse and zebrafish *Pdgfra* mutants can cause heart defects^19–21^. In zebrafish, ethanol has been shown to interact with *Pdgfra*, causing apoptosis of the neural crest cell precursors of the face in mutants and heterozygotes^21^. This study also demonstrated that the interaction was conserved in humans and noted an increase in gross cardiac defects in ethanol-treated zebrafish mutants (McCarthy *et al*.^21^). Collectively, these studies suggest that variants in the *PDGF* pathway could modulate the genesis of congenital heart defects in humans.

Using data from 806 OHD case and 996 control families enrolled in the NBDPS with birth dates between 1997 and 2011, we sought to evaluate interactions between 102 variants in the *PDGF* family of genes (*PDGFRA*, *PDGFRB* and *PDGFB*) and periconceptional alcohol exposure on the risk for OHDs. Maternal and infant genotypes were both investigated, since both are shown to alter the intrauterine environment and simultaneously contribute to OHD development^6,22,23^.

## Results and Discussion

### Study Sample

The final analytic sample includes 3422 individuals from 806 case-parental triads and 996 control-mother dyads. The demographic and lifestyle characteristics of case and control mothers are summarized in Table 1. Case mothers were slightly older (27.8 years vs. 26.8 years) and more highly educated than control mothers (more than high school: 59% cases vs. 52% controls). There were more Caucasian mothers in the case group (63% cases vs. 56% controls) and more Hispanic mothers in the control group (24% cases vs. 32% controls).

**Table 1:** Maternal characteristics.

	Case (N = 806)	Control (N = 996)
Maternal age at delivery, mean (SD)	27.8 (5.8)	26.8 (5.8)
Maternal race, n (%)		
Caucasian	506 (63%)	558 (56%)
African American	42 (5%)	59 (6%)
Hispanic	194 (24%)	323 (32%)
Others	64 (8%)	56 (6%)
Maternal education, n (%)		
0–11 years	123 (15%)	200 (20%)
Completed high school	205 (25%)	269 (27%)
1–3 years of college	235 (29%)	283 (28%)
4 years college or more	241 (30%)	244 (24%)
Alcohol exposure I (B1-M3), n (%)		
Binge drinkers^a^	126 (16%)	148 (15%)
Non-binge drinkers^b^	667 (84%)	842 (85%)
Alcohol exposure II (B1-M3), n (%)		
Binge drinkers	126 (20%)	148 (19%)
Non-drinkers^c^	497 (80%)	634 (81%)

SD = standard deviation.

^a^Binge drinkers are mothers who had four or more drinks on one occasion during the exposure window (i.e. from one month prior to conception (B1) to three months after conception (M3)).

^b^Non-binge drinkers are mothers who had less than four drinks on one occasion or did not have any alcohol consumption during the exposure window.

^c^Non-drinkers are mothers who did not have any alcohol consumption during the same exposure window.

### Binge Drinkers vs. Non-binge Drinkers

Among 806 case mothers, 126 (16%) were identified as binge drinkers (see definition in Data Collection); whereas among 996 control mothers, 148 (15%) were identified as binge drinkers. Binge drinking alone was not found to be significantly associated with OHD risk. The odds of giving birth to an infant with OHD among binge drinkers was estimated to be 1.07 times that among non-binge drinkers (95% CI: 0.83 to 1.39, p = 0.5847). Thus, when genotype is not considered there appears to be no relationship between drinking pattern and OHD risk.

To investigate the interaction between genetic variant and binge drinking, a log-linear model was fitted for each variant. The final analysis included 43 variants in the *PDGFRA* gene, 58 variants in the *PDGFRB* gene, and one variant in the *PDGFB* gene. The maternal genotype of a variant in the *PDGFRA* gene (rs869978) was identified to associate with the risk of OHDs via an interaction with maternal binge drinking at a nominal significance level (p = 0.0373). Binge drinkers carrying CT genotype for rs869978 had an increased risk of having infants with OHDs compared to those carrying CC genotype (genetic relative risk (GRR): 1.49; 95% CI: 1.05 to 2.12). In contrast, no significant genetic effect was found among non-binge drinkers (GRR: 0.99, 95% CI: 0.84 to 1.17). Under the assumption of multiplicative risk of alleles (see Statistical Method for details), the GRRs for comparing maternal TT genotype to maternal CC genotype of variant rs869978 were estimated to be 2.22 (95% CI: 1.10, 4.49) among binge drinkers and 0.98 (95% CI: 0.71, 1.37) among non-binge drinkers, respectively. Collectively, these findings suggest a maternal genotype-case phenotype interaction with the maternal T allele of variant rs869978 increasing the risk of OHDs occurring in offspring whose mothers were exposed to binge drinking. After adjustment for multiple testing, the above association was not statistically significant.

### Binge Drinkers vs. Non-drinkers

Non-binge drinkers included both mothers who had less than four drinks on one occasion and those who did not have any alcohol consumption during the exposure window. To avoid biased results due to a mixed group of non-binge drinkers, we excluded those mothers who had less than four drinks on one occasion, and conducted analyses to compare binge drinkers to non-drinkers. Mothers who were binge drinkers were approximately 1.09 times more likely to give birth to an infant with OHD than those who are non-drinkers (95% CI: 0.83 to 1.42, p = 0.5416). Table 2 summarizes findings from this analysis. These results are consistent with those above showing no relationship between binge drinking and OHDs in this cohort in the absence of genotype.

**Table 2:** The significant maternal and infant SNPs interacting with maternal alcohol exposure (binge drinkers vs. non-drinkers).

Maternal genotype							
Chromosome	Gene	SNP	SNP type	Genotype	Non-drinkers GRR (95% CI)	Binge-drinkers GRR (95% CI)	Interaction p-value^a^
4	PDGFRA	rs869978	Intron	CC	Reference	Reference	0.0242
				CT	0.96 (0.79, 1.17)	1.51 (1.06, 2.15)	
				TT	0.92 (0.62, 1.37)	2.28 (1.12, 4.62)	
Infant genotype							
Chromosome	Gene	SNP	Type	Allele	Non-drinkers RR (95% CI)	Binge-drinkers RR (95% CI)	Interaction p-value^b^
4	PDGFRA	rs2291591	Intron/Missense	CC	Reference	Reference	0.0357
				CT	1.07 (0.75, 1.53)	0.41 (0.18, 0.94)	
				TT	1.14 (0.56, 2.34)	0.17 (0.03, 0.88)	
4	PDGFRA	rs2228230	Synonymous	GG	Reference	Reference	0.0380
				AG	1.04 (0.84, 1.29)	0.58 (0.34, 0.97)	
				AA	1.08 (0.71, 1.66)	0.34 (0.12, 0.94)	
4	PDGFRA	rs1547904	Intron	CC	Reference	Reference	0.0385
				CT	1.04 (0.83, 1.28)	0.58 (0.34, 0.97)	
				TT	1.08 (0.69, 1.64)	0.34 (0.12, 0.94)	
4	PDGFRA	rs869978	Intron	CC	Reference	Reference	0.0395
				CT	1.06 (0.86, 1.29)	0.65 (0.42, 1.00)	
				TT	1.12 (0.74, 1.66)	0.42 (0.18, 1.00)	

GRR = genetic relative risk.

CI = confidence interval.

^a^Maternal interaction p-value = p-value for the interaction between disease status, maternal genotypes and maternal alcohol exposure.

^b^Infant interaction p-value = p = value for the interaction between disease status, infant genotypes and maternal alcohol exposure.

Similar to the comparison between binge drinkers and non-binge drinkers, a nominally significant genetic effect was identified for maternal variant rs869978 for binge drinkers versus non-drinkers (p = 0.0242). Here again, the maternal T allele associates with an increased occurrence of OHDs in infants exposed to prenatal ethanol.

The infant genotypes also interacted with ethanol in this set of analyses. The infant genotypes of four variants (i.e. rs2291591, rs2228230, rs1547904, and rs869978) in the *PDGFRA* gene were found to associate with the risk of OHDs through an interaction with maternal binge drinking at a nominal significance level (p = 0.0357, 0.0380, 0.0385, and 0.0395 respectively). In each instance, the alternative allele associates with reduced occurrence of OHDs in infants with prenatal alcohol exposures, but not for those without exposure. For example, among binge drinkers, infants carrying the CT genotype for rs2291591 had a decreased risk of OHDs compared to those carrying the CC genotype (GRR: 0.41; 95% CI: 0.18 to 0.94). In contrast, no significant difference was found among non-drinkers (GRR: 1.07, 95% CI: 0.75 to 1.53). Similar effects were detected for the other three variants in the same gene. Variants rs2291591, rs2228230, and rs1547904 were determined to be in high linkage disequilibrium (LD; D^’^ = 0.93), suggesting that these three variants are measuring the same genetic effect. After adjustment for multiple testing, none of the associations reported above were statistically significant.

Among 102 variants in the *PDGF* family, multiple variants in the *PDGFRA* gene were identified to potentially influence risk of OHDs by interacting with maternal binge drinking. None of the variants were found to significantly impact the risk of OHDs among non-binge drinkers or non-drinkers. Our findings indicate that there may be both maternal and infant genotypes that correlate with OHDs. Given that previous research has reached mixed conclusions^9–14^, our work suggests that part of these inconclusive results could be due to gene-ethanol interactions masking the overall effect of ethanol. Because the primary goal of our study is gene discovery, and the sample size was relatively small for detecting a small to moderate interaction effect, we have used a nominal threshold for statistical significance. If a Bonferroni correction was used to adjust for multiple testing, none of the SNPs would reach statistical significance. Furthermore, no adjustment was made for confounding effects from other covariates when fitting the model. Although the estimated infant genetic effects were robust in the presence of population stratification or other confounders such as maternal age and smoking, the estimated maternal genetic effects may be susceptible to other confounding effects. Nevertheless, NBDPS is the largest population-based multi-site study of birth defects ever conducted in the United States (US), and we have utilized all samples available to date. The current study is among the first ones to explore the joint effects of both genetic variants and maternal binge drinking on the risk of OHDs. Our findings need to be replicated in follow-up studies when additional independent samples become available.

Our findings suggest that the maternal genotype of rs869978 may increase the risk of OHDs in children exposed to alcohol during the periconception period. When considering the infant genotype, our study identified four variants within *PDGFRA* that nominally associated with reduced occurrence of OHDs following alcohol exposure during the periconceptional period. The four identified variants consist of two intron variants, one synonymous variant, and one variant with a combination of intron and missense (Table 2). These variants are in LD, so it is unclear the extent to which each individual variant may contribute to overall risk for OHDs. It is of interest that maternal genotype of rs869978 also associated with OHDs following periconceptional alcohol exposure. However, this same variant nominally associated with increased risk for OHD when maternal. The reasons for these differing associations are unclear, and may be related to different roles for *PDGF* signaling programs between the mother and the developing fetus^24,25^; indeed, the functional consequences of any of these variants are unknown.

However, the function of *Pdgfra* has been extensively studied in animal models and may help provide insight into possible roles for these genotype-ethanol interactions. Platelet-derived growth factors (*PDGFs*) and their receptors (*PDGFRs*) are involved in many cellular processes such as migration, survival and proliferation, and they are critical during developmental processes^26,27^. Cardiac development requires *Pdgfra* in both mouse and zebrafish^28,29^. In mouse *Pdgfra* mutants, cardiac neural crest cells have migratory defects^20^; and *Pdgfrb* double mutants have more extensive cardiac neural crest cell migration deficits^19^. In zebrafish the migration of mesoderm-derived cardiac progenitors requires *Pdgfra* function (Bloomekatz *et al*., 2017). Cardiac neural crest cells do populate the heart in zebrafish^15,16^, although it’s unclear if these cells require *Pdgfra* function. How ethanol may interact with *PDGFRA* in humans remains an intriguing question that may be elucidated by further analyses in zebrafish.

## Materials and Methods

### Ethics Statement

The study was approved by the University of Arkansas for Medical Sciences’ Institutional Review Board. The NBDPS was approved by the Institutional Review Boards at the Centers for Disease Control and Prevention and all NBDPS institutions. All study subjects gave informed consent. For minors in legal custody of their parents, informed written consent was obtained from their legal guardian for DNA collection. All methods were performed in accordance with the relevant guidelines and regulations.

### Study Population

Families were recruited for the NBDPS, a population-based case-control study designed to identify infants with major structural birth defects and evaluate genetic and environmental factors associated with the occurrence of birth defects^30,31^. The families included in the current study were identified through population-based birth defects surveillance systems in the states of Arkansas, California, Georgia, Iowa and Texas. A total of 4,101 individuals from 873 case-parental triads and 1,084 control-mother dyads were included. Cases were singleton live-born infants with OHDs. Both right-sided and left-sided OHDs included hypoplastic left heart syndrome, tricuspid atresia, pulmonary valve atresia, coarctation of the aorta, interrupted aortic arch types A and C, aortic stenosis, pulmonary valve stenosis, and Ebstein’s anomaly^32^. Cases where the pregnancy was affected by a known single gene disorder, chromosomal abnormality, or syndrome were excluded. A classification system developed for NBDPS, which incorporated three dimensions of cardiac phenotype, cardiac complexity, and extracardiac anomalies, was used to classify cases^32^. Controls were singleton live-born infants without any major structural birth defects. The mothers of all participating families completed interviews.

### Data Collection

Information regarding multiple maternal exposures and lifestyle factors hypothesized to impact developing embryos was obtained from a 1-hour telephone interview with each case and control mother. The interviews were conducted by female interviewers using a computer assisted telephone questionnaire^30,31^. All study materials were available in English and Spanish^30,31^. For the current study, we focused on interview information regarding periconceptional alcohol exposure. Based on NIAAA criteria, binge drinkers are defined as mothers who had four or more drinks on one occasion during the exposure window (i.e. from one month prior to conception to three months after conception). We included one month prior to conception in the exposure window to include many mothers who may not know the exact date of conception and may unknowingly continue drinking during the critical period of embryogenesis as up to half of US pregnancies are unplanned^33,34^. Non-binge drinkers are mothers who had less than four drinks on one occasion or did not have any alcohol consumption during the exposure window. Non-drinkers are mothers who did not have any alcohol consumption during the exposure window. Once a mother completed the interview, a buccal-cell sample-collection kit with instructions was mailed to the family for collection of DNA from the child, mother and father^30,31^.

### Genotyping and Quality Assessment

From the genotyping results of the Illumina HumanOmni5Exome BeadChip, 275 variants from the *PDGF* family of genes (*PDGFRA*, *PDGFRB* and *PDGFB*) were selected for analysis. DNA was extracted from buccal cell samples using Puregene DNA purification reagents (Qiagen, Valencia, CA), Genotyping was performed using the HumanOmni5Exome BeadChip (Illumina, San Diego, CA). Genotype calls were generated using GenCall, Illumina’s proprietary genotyping algorithm. A total of 4,101 individual samples were genotyped, and 679 samples were removed from analysis due to study ineligibility (n = 16), low genotype call rates (n = 663), resulting in an analytic sample consisting of 3,422 individuals from 806 case-parental triads and 995 control-mother dyads. We excluded 43 non-polymorphic variants, 17 variants with no-call rates > 5%, and 113 variants with minor allele frequencies < 1%. After the quality assessment, the analytical data had 102 variants in total.

### Statistical Method

Summary statistics were expressed as means (standard deviation) for continuous variables, and counts (percentage) for categorical variables. The means of continuous variables were compared using the two-sample t-test, while the proportions of categorical variables were compared using the chi-squared test. Because both case-parental triads and control-mother dyads were enrolled and genotyped in our study, a hybrid design where case-parental triads were augmented by control-parental dyads, provided optimal power for studying both the maternal and infant genetic effects^35,36^. Based on the hybrid design, the following log-linear (Poisson) model was fitted for each variant^6,35–37^.

1 $$\begin{array}{c}ln[E(count|M,\,F,\,C,\,D,\,E)]\\ \,\,\,\,\,=\,\mathrm{ln}({\mu }_{j}+{\delta }_{j}{I}_{(E=1)})+\gamma {I}_{(D=1)}+{\beta }_{1}{I}_{(D=1)}{I}_{M}+{\beta }_{2}{I}_{(D=1)}{I}_{C}+{\beta }_{3}{I}_{(D=1)}{I}_{(E=1)}{I}_{M}\\ \,\,\,\,\,\,+{\beta }_{4}{I}_{(D=1)}{I}_{(E=1)}{I}_{C}+ln(off),\end{array}$$

where *μ*_*j*_, *j* = 1. …, 6 are stratum parameters indicating six possible mating types under the assumption of mating symmetry; *μ*_*j*_ + *δ*_*j*_ are corresponding stratum parameters for ethanol-exposed families; *I*_(*E*=1)_ equals to 1 for ethanol-exposed families and 0 otherwise; *I*_(*E*=1)_ equals to 1 for case families and 0 for control families; *I*_*M*_ is an indicator for maternal genetic effects and equals the number of copies of the variant allele (0, 1, or 2) carried by the mother; and *I*_*C*_ is the corresponding indicator for infant genetic effects. Indicators *I*_*M*_ and *I*_*C*_ are defined in this way to assume multiplicative (i.e. log-additive) risk per allele. Under this assumption, the relative risk of carrying two copies of the allele is the square of the relative risk of carrying one copy of the same allele, with zero copies as the reference. This model specification provides population-based inferences related to both maternal and infant genotypes, and additional family-based evidence related to infant genotypes that is robust to population admixture, and allows estimation of gene-ethanol interactions and inclusion of incomplete families using the Expectation-Maximization (EM) algorithm for handling missing genotypes. Comparisons were made between families of mothers who were binge drinkers vs. non-binge drinkers, and between families of mothers who were binge drinkers vs. non-drinkers. Statistical significance was assessed as p-value ≤ 0.05. We used R statistical software v3.2.3 (R Foundation for Statistical Computing, Vienna, Austria) for computing summary statistics, and LEM^38^ statistical software to fit log-linear models with incomplete family genotype data. Patterns of linkage disequilibrium (LD) between important variants from the same gene were constructed using HaploView 4.2^39^.

As discussed comprehensively elsewhere, the log-linear model being used here has a few unique strengths and limitations^35,36^. It is worthwhile to note that the infant genetic effects are estimated using the case-parents triads. By conditioning on parental genotypes, the transmissions of alleles to affected offspring can be tested against the null hypothesis of random transmission (i.e. Mendel’s law). Such a family-based association test strategy is robust to population stratification and other confounding factors, such as maternal age and smoking. However, the maternal genetic effects uses a population-based association test strategy by contrasting between case mothers and control mothers, and thus, are susceptible to unknown confounding factors. Meanwhile, the log-linear model partition family units into strata of gene-by-environment combinations, and does not allow estimation of “main effect” for additional covariates.
